# Teratogenic effects of silymarin on mouse fetuses

**Published:** 2016

**Authors:** Mahbobe Gholami, Seyed Adel Moallem, Mohammad Afshar, Sakineh Amoueian, Leila Etemad, Gholamreza Karimi

**Affiliations:** 1 *Department of Pharmacodynamics and Toxicology, School of Pharmacy, Mashhad University of Medical Sciences, Mashhad, Iran*; 2 *Pharmaceutical Research Center, Mashhad University of Medical Sciences, Mashhad, Iran*; 3 *Department of Anatomy, Birjand University of Medical Sciences, Birjand, Iran*; 4 *Department of Pathology, Imam-Reza Hospital, Mashhad University of Medical Sciences, Mashhad, Iran*; 5 *Pharmaceutical Research Center, Pharmacy school, Mashhad University of Medical Sciences, Mashhad, Iran*

**Keywords:** *Silybum marianum*, *Silymarin*, *Mouse fetus*, *Teratogenicity*

## Abstract

**Objective:**

*Silybum marianum* has been used for centuries in herbal medicine for treatment of liver diseases. Currently, there is no data available on the possible effects of silymarin on fetal development. This study aimed to investigate the teratogenic effect of silymarin on BALB/c mice fetuses.

**Materials and Methods:**

A total of 40 pregnant mice were divided into 4 groups of 10 mice each. Three groups received silymarin at three different doses of 50, 100 and 200 mg/kg/day during gestational days (GDs). The control group received normal saline and tween (solvent). Dams were sacrificed on GD 18 and all fetuses were examined for gross malformations, size and body weight. Malformed fetuses were double stained with alizarin red and alcian blue.

**Results:**

Silymarin administration at all doses resulted in reduction of the mean fetal body weights. The abnormalities included limb, vertebral column and craniofacial malformations. Craniofacial malformations were the most common abnormalities, but they were not observed in a dose-dependent manner. The percentage of fetal resorption significantly increased (up to 15%) in all treatment groups.

**Conclusion:**

Based on our results, silymarin, especially at high doses can lead to fetal resorption, intrauterine growth retardation and limb, vertebral column and craniofacial abnormalities. More precise studies should be conducted about the teratogenic effects of herbal medicine investigating the underlying mechanisms. Thus, caution should be taken when administering *S. marianum *to pregnant woman.

## Introduction

*Silybum marianum* (milk thistle) has been used for centuries in herbal medicine to treat liver disease. In 1968, a flavonolignan complex in milk thistle fruit was isolated and named silymarin. Silymarin is largely responsible for the medical benefits attributed to *S. marianum* and is mainly composed of silibinin (also called silybin) and other components such as isosilybin, silydianin and silychristin. Silibinin, the most active ingredient of silymarin, is the most well-known hepato-protective agent (Karimi et al., 2011 ▶).

*S. marianum* extract is water insoluble and usually administered orally as a standardized extract in encapsulated form (best formulation comprises 70-80 % silymarin). It is moderately absorbed (23-47 %) from the gastrointestinal tract. The maximum plasma concentration is achieved in approximately 1-2 hr after oral dosing of *S. marianum* extract in human (Polyak et al., 2013 ▶). Silymarin is primarily excreted through the bile while some of it is cleared via the kidneys with a clearance half-life of 6-8 hr. Silymarin and more specifically silibinin, can block the binding of potential hepatocellular toxins to the outer surface of the cells and directly relieve the hepatocytes (Campos et al., 1989 ▶; Muriel et al., 1992 ▶). Silymarin, as a strong free radical scavenger, has attracted intensive attention as it increases the formation of glutathione in hepatocytes (Sharma et al., 2008 ▶). It has been used in pregnant women with intrahepatic cholestasis (Giannola et al., 1985 ▶). It is used to prevent the effects of ethanol and cyclophosphamide on liver (Urban 2000 ▶; Ahmadi-ashtiani et al., 2010 ▶; Mahabady et al., 2011 ▶) and also has several health benefits against various liver conditions such as cirrhosis, hepatitis and fatty liver (Kaur et al., 2011 ▶).

Although no serious adverse effects, even at high doses, have been reported by German Commission E, many studies have reported heart burn, stomach upset and transient headaches, but none of these symptoms were due to supplementation with silymarin (Fraschini et al., 2002 ▶).

Despite wide use of *S. marianum* and its active components in traditional and modern medicine, there is not enough information on this herb; therefore, we investigated silymarin potential embryotoxicity following exposure during organogenesis, in BALB/c mice fetuses.

## Materials and Methods

**Materials**

Silymarin and tween were purchased from Sigma Company (Germen). Alizarin red and alcian blue were purchased from Merck (Darmstadt, Germany). 

**Animal treatment**

The present experimental study was carried out using 40 virgin female BALB/c mice (20-30g and approximately 2 months old). Mice were obtained from Avicenna Research Institute of Mashhad University of Medical Sciences, Mashhad, Iran. The mice were kept at room temperature of 23±2 ˚C with 12-hr/12-hr light/dark cycles and had unlimited access to food and water. The protocol of this study was approved by the Animal Care and Ethics Committee of Mashhad University of Medical Sciences, Mashhad, Iran.

One male was caged with two females over night and they observed for the presence of vaginal plug in the next morning. The day with the presence of the vaginal plug was considered gestational day (GD) 0. The mice were randomly divided into four groups. Three groups received silymarin at doses of 50, 100 and 200 mg/kg/day (group I, II and III) via intraperitoneal (IP) injection, during GD6–GD15 (organogenesis period). Doses were selected based on previous animal studies (Kasim et al., 2009 ▶; Malekinejad et al., 2011 ▶). The control group received normal saline and tween (solvent) via the same route at an equivalent volume (0.5 ml). 

**Maternal observation**

Maternal body weights were investigated throughout the pregnancy period. All groups were observed daily for mortality, morbidity and general appearance and behavior. Maternal body weight gain was calculated by subtracting the weight of pregnant mice on GD0 from that of GD18.

**Fetus observation and staining**

On GD 18, pregnant mice were sacrificed under ether anesthesia and cesarean section was performed. Fetuses were removed from uterine and after cutting the umbilical cord, each uterus was examined individually for embryonic resorption. All fetuses were assessed for external malformations, size (crown-rump length) and body weight. External/macroscopic malformations (exencephaly, cleft palate, abdominal hernia, polydactyl, open eyelid, etc.) as well as growth limitation were checked under a stereomicroscope (Olympus SZX, Japan). Malformed fetuses were then selected and stained by a specific double staining. For skeletal staining, after the fetuses were skinned and eviscerated, specimens were fixed in 99% ethanol for three days then for two days in acetone. Then, samples were placed in a mixture of 0.01% % alcian blue and 0.005% alizarin red S in ethanol and glacial acetic acid. After rinsing with tap water, specimens were macerated in 2% KOH for 2 days. Finally, cleaning of soft tissues was carried out by soaking in a graded KOH: glycerine series over the next week until in 100% glycerine (Kimmel et al., 1981 ▶; Afshar et al., 2010 ▶).

**Statistical analysis**

Fetal body weight and crown-rump length were reported as mean±SEM. Following ANOVA, Tukey test was done to compare differences between control and each experimental group. Concerning the frequency of absorbed and live fetuses, external malformation differences between the control and each experimental group were tested using Fisher’s direct probability test and when the frequency of each category was 5 or more, the Chi-Square test was used. The statistical analysis was carried out using SPSS software (Ver. 17). Differences were considered significant at p<0.05.

## Results

**Maternal observation**

No death occurred among the pregnant or virgin mice and all of the mothers were alive at the time of cesarean section. As shown in Table 1, there was no significant difference in maternal body weight gain among the groups. During the pregnancy period there were no notable changes in food and water intake and behavior signs among treated and control groups.

**Table 1:** Effect of different doses of silymarin on BALB/c mice fetuses

**Group III**	**Group II**	**Group I**	**Control**	
10	10	10	10	**Dams (Pregnant mice) (No)**
18.03±0.94	19.83±1.06	19.03±0.97	20.04±1.02	**Maternal weight gain, Mean ±SEM**
119	125	121	133	**Fetuses examined, No**
101(84.88)	111(88.8)	115(95.04)	133(100)	**Live fetuses, No (%)**
18(15.12)*	14(11.2)*	6(4.95)*	0	**Resorbed fetuses, No (%)**
0.59±0.21*	0.63±0.27*	0.69±0.24*	1.13±0.11	**Fetal body weight , Mean± SEM(g)**
17.01±2.23*	17.11±2.61*	17.23±2.19*	22.90±2.03	**Fetal length, Mean ± SEM(mm)**

IP: intraperitoneal; GD: gestational day.

Group I, II and III received IP silymarin 50, 100 and 200 mg /kg/day during GD6–GD15, respectively. Control group received normal saline plus tween.

* p< 0.05 compared to the control group.

**Fetal resorption in the experimental and control groups**

According to the obtained results, injection of silymarin resulted in significant increase in fetal resorption (p<0.05). The frequency of fetal resorption was obtained 4.95%, 11.2% and 15.12% in groups I, II and III, respectively. No fetal resorption was seen in the control group. The prevalence of resorption sites rose with increasing doses (Table1).

**Growth indicators in fetuses exposed to silymarin**

Silymarin administration at doses of 50, 100, and 200 mg/kg reduced mean fetal body weight and crown-rump length as compared to control group (p<0.05) (Table 1). A large number of the embryos in the experimental groups had growth retardation (Table 2). The growth retardation increased with increasing doses. No growth retardation was found in the control group.

**Table 2:** External malformations in BALB/c mice fetuses exposed to different doses of silymarin

**Group III**	**Group II**	**Group I**	**Control**	
10	10	10	10	**Litters ** **(No)**
101	111	115	133	**Live fetuses** **(No)**
0	3(2.7)	0	0	**Open eyes, ** **No (%)**
3(2.97)	2(1.8)	3(2.6)	0	**Limbs deformities,** **No (%) **
5(4.95)*	5(4.5)*	4(3.47)*	0	**Vertebral deformity, ** **No (%) **
7(6.93)*	9(8.1)*	8(6.95)*	0	**Mandibular hypoplasia and Open mouth, ** **No (%)**
0(0)	2(1.8)	2(1.73)	0	**Maxillary hypoplasia, ** **No (%) **
19(18.81)*	15(13.51)*	11(9.56)*	0	**Growth retardation, ** **No (%) **
2(1.9)	2(1.8)	3(2.6)	0	**calvarial deformity, ** **No (%)**

IP: intraperitoneal; GD: gestational day.

Group I, II and III received 50, 100 and 200 mg /kg/day of IP administration of silymarin during GD6–GD15, respectively. Control group received normal saline plus tween.

* p< 0.05 compared to the control group.

**Abnormalities in fetuses exposed to silymarin**

The teratogenic effects of silymarin mainly included craniofacial malformations, growth retardation, vertebral deformity and limb defects.

**Craniofacial malformations**

**Figure 1.**
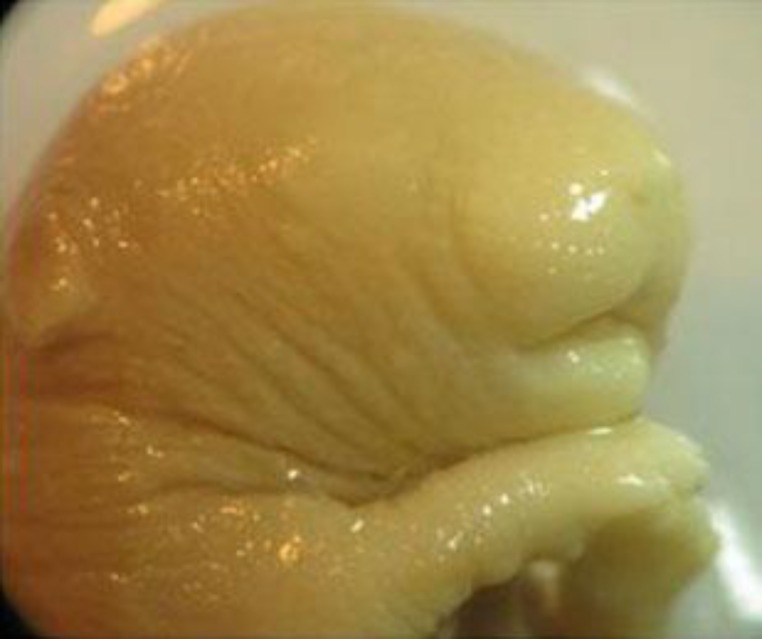
A fetus with mandibular hypoplasia deformities and open mouth from experimental group III that was treated with 200 mg/kg/day of silymarin

**Figure 2:**
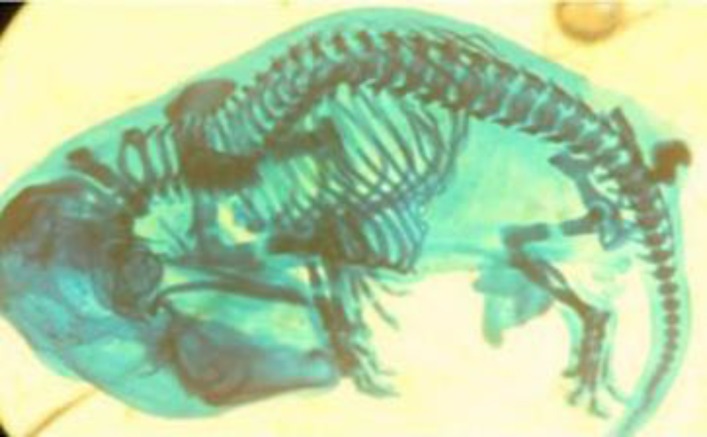
A fetus with mandibular hypoplasia deformities after skeletal staining from experimental group II, that was treated with 100 mg/kg/day silymarin

Craniofacial malformations were the most common abnormalities which were observed in fetuses exposed to silymarin. These malformations were mainly appeared as open mouth and mandibular hypoplasia (Figures 1 and 2). The obtained incidence were 6.95%, 8.1% and 6.93% in groups I, II and III, respectively (that were significantly different from the control group (p<0.05). Mandibular hypoplasia and open mouth had a higher incidence and maxillary hypoplasia was at low incidence level. There were no significant differences in calvarial deformity and maxillary hypoplasia among groups (Table 2).

**Vertebral deformities**

**Figure 3:**
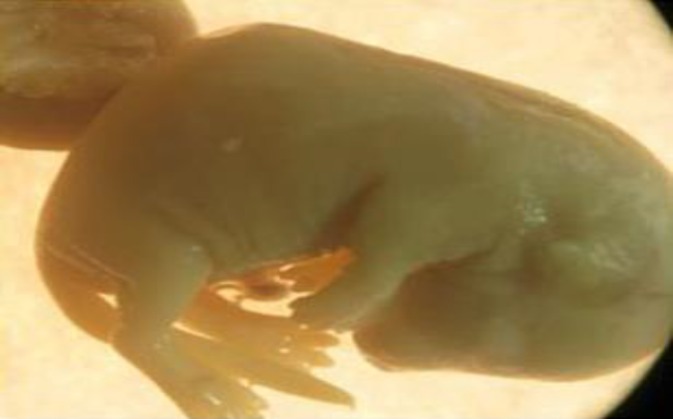
A fetus skeleton with kyphotic body from experimental group I that was treated with 50 mg/kg/day of silymarin

**Figure 4:**
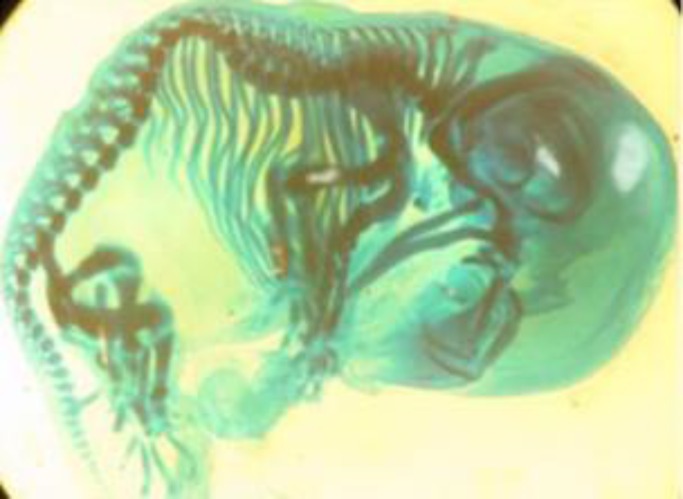
A fetus skeleton (stained with alizarin red S and alcian blue) with scoliosis from experimental group III that was treated with 200 mg/kg/day silymarin

Vertebral deformities determined as deviations in normal curvatures and kyphotic body. The prevalence of any vertebral deformity was estimated to be 3.47, 4.5 and 4.95% in groups I, II and III, respectively (Figures 3 and 4). There were significant differences in the incidence of vertebral deformities among treated and control groups (Table 2).

**Limb defects**

Low incidence of limb deformities were observed in groups I, II and III (2.6, 1.8 and 2.97%, respectively) which were not significantly different from the control group. These deformities included malrotation and delayed development in upper or lower limbs.

## Discussion

In recent years,* Silybum marianum* (milk thistle) extracts have been widely used to protect the liver cells against toxins. There is little information about silymarin side effects on fetal development. The results of this study showed that silymarin administration at doses of 50, 100 and 200 mg/kg during organogensis can cause fetal resorption and growth retardation. The intensity of alizarin red staining was lower in bones of growth-retarded fetuses including cranial, vertebral, metacarpal, metatarsal and phalanges in the presence of silymarin. Moreover, it may induce some malformations including craniofacial, vertebral and limb defects, in fetuses.

Many studies have mentioned that the anti-tumor effects of silibin are related to inhibition of DNA synthesis, cell proliferation and apoptosis induction accompanied by modulation of p53 (Mallikarjuna et al., 2004 ▶). Silymarin has been shown to exert multiple effects on cancer cells, including inhibition of both cell proliferation and migration in colon carcinoma cells (HT-29) (Woo et al., 2014 ▶).

On the other hand, excessive cell death is considered as one of the most important events corresponding to the intrauterine growth restrictions and fetal death (Torchinsky et al., 2005 ▶). Although apoptosis may be necessary to eliminate cells with DNA damage, it must also be tightly regulated in order to prevent inappropriate loss of normal cells. Programmed cell death in mammalian blastocyst is seen up to the next normal development stages. Both parts of the blastocyst (inner cell mass and trophectoderm) undergo apoptosis during the normal development; however, these parts have different sensitivities toward factors which cause apoptosis. Deviation in normal apoptosis in blastocyst may cause impaired fetal maturity and embryo death in the early stages. Apoptosis also plays a key role in the formation of the embryonic and extra-embryonic structures in the later stages of normal embryonic development. Apoptosis is considered as an important factor in fetal abnormalities and many teratogens act through this mechanism (Metcalfe et al., 2004 ▶; Torchinsky et al., 2005 ▶). Significant increase in resorption and growth retardation was observed in the fetuses that were exposed to silymarin which may be related to silymarin apoptotic activity (Malekinejad et al., 2011 ▶; Etemad et al., 2015 ▶).

The cytoprotective effects of silymarin are mainly attributable to antioxidant and anti-inflammatory properties (Pradhan et al., 2006 ▶). Silymarin like non-steroidal anti-inflammatory drugs (NSAIDs), inhibits cyclooxygenase enzymes (Ahmadi-Ashtiani et al., 2012 ▶), and decrease prostaglandin synthesis (Urban 2000 ▶). It is noticeable that NSAIDs may have side effects on bone and cartilage resorption (Gilroy et al., 1998 ▶; Nwadinigwe et al., 2007 ▶). It is interesting that malformation observed in our study was so similar to the pattern of malformations that are produced by many NSAIDs. It is indicated that oral administration of DuP-697 (a member of the diaryl heterocycle group of selective COX-2 inhibitors) to pregnant Wistar rats, during gestational days, induces higher incidence of ossification reduction of the supra occipital, hyoid, and metatarsal bones as well as developmental variations of the vertebral bodies in rat fetuses (Burdan et al., 2003 ▶). In the present study, vertebral malformations, limb defects and calvaria deformity were observed in the fetuses that were exposed to silymarin. In another study, gavage administration of celecoxib to pregnant Wistar rats during gestational days (day 6- day 20) caused some congenital malformations including kyphotic body, uni and bilateral malformation of both fore and hind limb and also a significant decrease in mandibular and limb bones ossification (Badawy et al., 2011 ▶). To evaluate the effect of diclofenac, embryos were exposed to various concentrations of diclofenac by using a whole rat embryo culture model. The results demonstrated that embryos in diclofenac-treated groups had a significantly lower total morphological score as compared to the control group and diclofenac exerted direct teratogenic effects on rat embryos (Chan et al., 2001 ▶). Non-selective cyclooxygenase inhibitors (ibuprofen, piroxicam, and tolmetin) administration to pregnant rats from the 8th gestational day to the 7th lactational day affected pups growth and influenced mineralization of the lumbar vertebrae (Burdan et al., 2011 ▶).

One of the common outcomes of NSAIDs consumption during pregnancy is the intrauterine growth retardation and decreased weight and crown-rump length of fetuses (Sorní et al., 2005 ▶; Fukushima et al., 2007 ▶; Burdan et al., 2009 ▶) which were also observed in our study. Therefore, the inhibition of cyclooxygenase may be a likely mechanism of silymarin’s teratogenicity effect in mouse fetuses. 

In conclusion, silymarin, especially at high doses can lead to embryo resorption and intrauterine growth retardation. Furthermore, it can induce some kind of malformations in the fetuses such as craniofacial, vertebral and limb defects. Therefore, it is suggested that great caution should be taken when prescribing silymarin during pregnancy and further investigations to reveal the underlying mechanisms should be performed. 

## Note

*Please cite this paper as: *

Gholami M, Etemad L, Moallem SA, Afshar M, Karimi Gh. Teratogenic effects of silymarin on mouse fetuses. Avicenna J Phytomed, 2016; 6 (5): 542-549.
